# Cytokines and Their Genetic Polymorphisms Related to Periodontal Disease

**DOI:** 10.3390/jcm9124045

**Published:** 2020-12-14

**Authors:** Małgorzata Kozak, Ewa Dabrowska-Zamojcin, Małgorzata Mazurek-Mochol, Andrzej Pawlik

**Affiliations:** 1 Chair and Department of Dental Prosthetics, Pomeranian Medical University, Powstańców Wlkp 72, 70-111 Szczecin, Poland; gosia-ko@o2.pl; 2 Department of Pharmacology, Pomeranian Medical University, Powstańców Wlkp 72, 70-111 Szczecin, Poland; dabewa@vp.pl; 3 Department of Periodontology, Pomeranian Medical University, Powstańców Wlkp 72, 70-111 Szczecin, Poland; malgorzata.mazurek@poczta.onet.pl; 4 Department of Physiology, Pomeranian Medical University, Powstańców Wlkp 72, 70-111 Szczecin, Poland

**Keywords:** periodontal disease, cytokines, polymorphism

## Abstract

Periodontal disease (PD) is a chronic inflammatory disease caused by the accumulation of bacterial plaque biofilm on the teeth and the host immune responses. PD pathogenesis is complex and includes genetic, environmental, and autoimmune factors. Numerous studies have suggested that the connection of genetic and environmental factors induces the disease process leading to a response by both T cells and B cells and the increased synthesis of pro-inflammatory mediators such as cytokines. Many studies have shown that pro-inflammatory cytokines play a significant role in the pathogenesis of PD. The studies have also indicated that single nucleotide polymorphisms (SNPs) in cytokine genes may be associated with risk and severity of PD. In this narrative review, we discuss the role of selected cytokines and their gene polymorphisms in the pathogenesis of periodontal disease.

## 1. Introduction

Periodontal disease (PD) is the second most common oral disease in the world and is still a significant and current public health problem [1]. Gingivitis is a mild and reversible form of PD, but it tends to turn into periodontitis [2]. PD begins with a disruption of bacterial homeostasis, leading to an increase in colonisation by periopathogenic microbes and inducing a host inflammatory response, which can lead to tissue destruction [3,4,5]. PD is caused by the accumulation of bacterial plaque biofilm on the teeth and the host immune responses that appear in the tissues in response to this [6]. The destructive process in supporting tissues involves ligaments and the alveolar bone [7,8]. Inflammatory and immune responses to oral microorganisms initiate the development of PD, which is considered one of the main reasons for tooth loss in developing and developed nations [9]. However, the inflammatory response of the periodontal tissues to infection is modulated by various environmental and genetic factors [10]. The pathogenesis of PD has not been completely defined. Many factors have an impact on the aetiology and pathogenesis of PD. It is believed that host genetic, microbiological, and environmental factors can determine the development of the disease [11,12]. There are many non-modifiable risk factors such as age, sex, race, and genotype, whereas modifiable risk factors include poor oral hygiene, specific perio-pathogens, smoking, obesity, diabetes, osteoporosis, stress, and socio-economic status. The heritability of periodontitis was assessed as 0.38 (95% CI, 0.34–0.43) in twin studies and 0.15 (95% CI, 0.06–0.24) in other family studies [13].

The interplay between smoking and genes in the risk of PD has been analysed in numerous studies. Cigarette smoking is a crucial environmental risk factor for PD development [14]. The risk of PD in smoking may be affected by polymorphisms of the N-acetyltransferase (NAT2) gene, which may alter the subject’s metabolism to produce more damaging smoking-derived xenobiotics [15]. Meisel et al. [15] have shown that the NAT2 genotypes could be the risk factors for PD. Severe chronic PD patients are mainly slow acetylators. Therefore, the slow acetylator phenotype may be related to a higher risk of PD. Moreover, nicotine is the main component of cigarette smoke and is one of its most pharmacologically active agents [16]. Another risk factor for PD could be polymorphisms of the cytochrome P450 CYP2A6 gene [17].

Genetic polymorphisms, especially in genes encoding molecules of the host defence system, such as cytokines, influence susceptibility to disease and its severity [18,19]. In periodontal tissues, immune cells (macrophages, lymphocytes), epithelial cells and pro-inflammatory cytokines including interleukin IL-1, IL-2, IL-6, IL-8, IL-10, IL-13, IL-17, IL-18, IL-35, matrix metalloproteinases (MMPs) and tumour necrosis factor-α (TNF-α) can generate immune responses [20,21]. The role of cytokines in the pathogenesis and severity of PD has been evaluated in several studies [21,22]. It has been proposed that the genetic factors of chronic periodontal disease (CP) are not numerous [23], but they are more significant in aggressive periodontal disease (AgP), as has been shown in familial aggregation research [24]. Two prime hypotheses have been proposed about the genetic basis of complex diseases. The first one is presented as the common disease/common variant hypothesis. [25]. It is believed that genetic variants that are common in the general population, but which individually have a weak impact are those that have the greatest effect on genetic susceptibility to complex disorders. When genetic components occur with a frequency of at least 1% in the population, they are called polymorphisms [26]. They contain single nucleotide polymorphisms (SNPs), which are very common in the genome and consist of the alteration of one nucleotide base for another. The second hypothesis is the common disease/rare variant hypothesis, in which the main contributors to susceptibility to complex diseases are infrequent variants (minor allele frequency < 1%) present in the genome [26].

Kornman et al. published one of the first research studies to discover genetic markers of PD [27]. The authors studied a few polymorphisms of the interleukin 1 (IL-1) gene and observed a correlation between IL-1 gene polymorphisms and the severity of PD in non-smokers, differentiating between patients with mild and severe PD. These interesting studies contributed to the next genetic research studies on PD, which used various methodological projects. The identification of genetic risk factors correlated with PD is significant for developing prevention and treatment plans for this disease [28,29].

Cytokines and their genetic polymorphisms may also play a role in the development of aggressive periodontitis which, before 1999, was classified as early-onset periodontitis (EOP), characterized by rapid attachment loss and bone destruction [30,31]. Cytokines activate several signalling pathways modulating osteoclastic activity and alveolar bone loss. Previous studies have shown that cytokine gene polymorphisms may be associated not only with the risk of periodontal disease but also may affect disease progression and its severity [32,33].

## 2. Cytokines Associated with Periodontal Disease

### 2.1. Interleukin 1

IL-1 plays significant role among interleukins in the pathogenesis of PD and many other inflammatory diseases [5,34,35]. IL-1 is involved in a number of processes that are important for initiating and sustaining an inflammatory response [36]. IL-1 plays an important role in the tissue destruction connected with PD due to its pro-inflammatory and bone-resorptive properties. Research has shown that polymorphisms in the genes encoding IL-1 might be related to greater PD severity [27,28,29,36,37]. He et al. suggested that the IL-1α −889C/T and IL-1β +3954C/T gene polymorphisms are related to the risk of peri-implantitis [37]. Kornman et al. [27] observed a correlation between polymorphisms in the genes encoding for IL-1α −889C/T and IL-1β +3953C/T and an elevated severity of PD. In a study by Mazurek-Mochol et al., there were no statistically significant associations between IL-1β gene +3954C/T and −511C/T polymorphisms and periodontal disease in smoking and non-smoking patients [38]. Da Silva et al. showed in a meta-analysis that the IL-1β +3953C/T polymorphism is related to elevated risk of chronic periodontitis in Caucasian and Asian populations [39] (Table 1).

Parkhill et al. suggested that the IL-1β gene +3953C/T polymorphism may be the risk factor for early-onset periodontitis in smoking Caucasian patients [41]. The authors of another study found the associations between IL-1α gene −889C/T and IL-1β gene +3953C/T polymorphisms and early-onset periodontitis in African American and Caucasian populations [56]. The meta-analysis by Chen et al. indicated a lack of association between the IL-1β gene +3953C/T polymorphism and aggressive periodontitis, regardless of ethnicity [57]. Additionally, in studies conducted in other populations, there was no significant association between the IL-1β gene +3953C/T polymorphism and aggressive periodontitis [58]. 

Puri et al. suggested the association between the IL-1α gene −889C/T polymorphism and aggressive periodontitis in the Indian population. The results of studies conducted in other populations indicate a lack of association between the IL-1α gene −889C/T polymorphism and aggressive periodontitis [59]. In the study by Tai et al., there were no significant associations between early-onset periodontitis and the IL-1α gene +4845C/T as well as IL-1β −511T/C, +3954C/T polymorphisms in the Japanese population [60]. In a meta-analysis, Feng et al. indicated that the IL-1α −889C/T polymorphism is connected with susceptibility to chronic periodontitis in African, European and American populations [40].

The specific genotype of the polymorphic IL-1 gene cluster is connected with the severity of PD and distinguishes individuals with severe PD from those with mild disease. Functionally, the specific PD-associated IL-1 genotype consists of a variant in the IL-1α gene that is related to high levels of IL-1 production [27]. Kornman et al. [27] noticed that 86.0% of severe PD patients are accounted for either by smoking status or by the IL-1 genotype. Similar results were found by McGuire and Nunn [61] and Laine et al. [62]. Patel et al. also observed that smokers with periodontitis demonstrated higher levels of IL-1β compared with non-smokers [63]. Other contradictory studies, such as that of Meisel et al. [64], found that the composite genotype shows a strong interaction with smoking, one of the established risk factors for PD, whereas non-smokers, even genotype-positive ones, are not at any raised risk. A similar contradictory study [37] on 132 PD patients who were age- and sex-matched with controls did not show any relationship between genotype and PD. This may be due to ethnic differences, the models used in these studies or the limited number of cases. Polymorphisms in the IL-1 gene cluster linked with PD [65] are observed in approximately 30% of the European population. Additionally, the prevalence is lower in the Chinese population (2.3%) and therefore the helpfulness of the genotypes of both IL-1α +4845C/T and IL-1β +3954C/T for determining susceptibility in Chinese patients is uncertain [66,67].

### 2.2. Interleukin 6

Interleukin 6 (IL-6) is a lymphocyte chemoattractant factor secreted by a wide variety of cells, such as lymphocytes, mast cells, eosinophils, and epithelial cells [68,69]. IL-6 plays a vital and important role in the pathogenesis of PD. This pro- and anti-inflammatory cytokine stimulates IL-2 receptor expression and the secretion of other pro-inflammatory interleukins [70]. Moreover, it blocks the synthesis of pro-inflammatory cytokines IL-1 and TNF-α [71]. IL-6 induces osteoclast differentiation and bone resorption and inhibits bone formation [72]. At low concentrations, IL-6 stimulates osteoclast formation from precursors, but at high concentrations, IL-6 mostly induces the activation of mature osteoclasts [72]. Apart from the influence of IL-6 on osteoclasts, research has also shown that IL-6 connects with the release and activation of MMPs, leading to the pathological breakdown of the extracellular matrix (ECM) in PD. Previous studies have shown elevated serum levels of IL-6 in patients with periodontal disease [71,72,73,74,75,76]. 

Regulation of IL-6 production is generally associated with genetic factors. There are many known promoter polymorphisms in the IL-6 gene: −597G/A, −572C/G and −174G/C [72]. The associations between these polymorphisms and PD were confirmed in candidate gene studies and meta-analyses. The SNPs −572G/C and −174G/C, which influence the expression of IL-6 and its serum level, were related to susceptibility to PD [42,76,77]. 

The effects of studies on the associations between these polymorphisms and various clinical forms of PD are contradictory [42,43,44,78,79,80,81,82,83,84,85,86]. Costa et al. [82] observed in the Brazilian population that the IL-6 −174G/C gene polymorphism may play a role in chronic periodontal disease. Tervonen et al. [75] have found that the IL-6 −174GG gene polymorphism may be associated with the development of PD in the Caucasian population. Additionally, other authors have suggested a correlation between the IL-6 −174GG allele and various forms of PD [79]. There are also other studies indicating no association between the IL-6 −174G/C polymorphism and PD in Caucasian and Asian populations [43,83,84,85]. The meta-analysis by Shao et al. that included nine studies (1093 patients with PD and 574 controls) found that the IL-6 −174G/C polymorphism is not associated with the risk of chronic periodontal disease, but the IL-6 −174G allele may increase the risk of aggressive periodontitis [42]. The meta-analysis by Zhu et al. that included 21 case-control studies suggests that the IL-6 −174GG genotype is associated with chronic periodontal disease in Brazilian and Caucasian populations [87].

These studies have indicated the association between the IL-6 −572GG genotype and periodontal disease. Holla et al., in a candidate gene study, have shown the association between the IL-6 −572GG genotype and chronic periodontal disease in the Caucasian population [43]. In the study by Komatsu et al., this polymorphism was associated with chronic periodontal disease in a Japanese population [77]. The study by Nibali et al. has suggested the association between the IL-6 −572GG genotype and aggressive periodontitis [44]. In the meta-analysis by Shao et al., the IL-6 −572GG genotype was associated with chronic periodontal disease and aggressive periodontitis [42]. The different results of the above studies may be due to different genetic predisposition to chronic periodontal disease and aggressive periodontitis, as well as differences in the pathogenesis of these two forms of periodontal disease.

### 2.3. Interleukin 8

Interleukin 8 (IL-8) is the chemokine involved in the pathogenesis of PD. This cytokine activates the migration of neutrophils in inflammatory regions [68]. The IL-8 gene is located on chromosome 4q13-q21 [46,88]. Previous studies have shown the association of IL-8 SNPs with PD [45]. Kashiwagi et al. have shown increased production of IL-8 in epithelial cells induced by oxidative stress [89]. Borilova Linhartova et al. indicated increased circulating levels of IL-8 in patients with diabetes mellitus and periodontal disease [90]. 

Patel et al. observed that smoking appears to have an inhibitory impact on chemokine production, reflected in a reduced amount of IL-8 in gingival crevicular fluid [63]. This reduction in IL-8 levels in smokers causes impaired neutrophil chemotaxis and migration in the periodontium, paving the way for the progression of PD.

Common polymorphisms have been reported in the IL-8 gene: −251A/T, +396G/T, −845T/C and +781C/T. Some studies have revealed a connection between IL-8 SNPs and PD susceptibility [47,48,91], while others failed to show an association of PD with IL-8 gene polymorphisms [92,93].

Scarel-Caminaga et al. have shown that the IL-8 +396TT genotype was associated with chronic periodontal disease in the Brazilian population [47]. In the study by Andia et al., the IL-8 −251A allele (rs4073) was significantly associated with increased risk of chronic periodontal disease in non-smoking Brazilian patients [48]. These authors have suggested a lack of association between this polymorphism and aggressive periodontitis [93]. This association was not confirmed by Kim et al., who suggested a lack of association between the IL-8 −251A/T (rs4073) polymorphism and PD in the Brazilian population [92]. Borilova Linhartova et al. also suggested a lack of association between IL-8 gene (rs4073, rs2227307, rs2227306, and rs2227532) polymorphisms and PD in the Czech population. The above differences may be due to ethnic differences and different pathogenesis of chronic periodontal disease and aggressive periodontitis [94].

### 2.4. Interleukin 10

IL-10 is a significant contributor to the pathogenesis of PD [95]. Several studies have presented that serum IL-10 levels in patients with CP are higher than in periodontally healthy individuals [48]. IL-10 suppresses the production of IL-1, TNF-α, IL-6, IL-8, IL-12, and IL-18 [96,97]. 

Interleukin 10 (IL-10) is a cytokine that is involved in the pathogenesis of PD [98]. In a clinical study, it was observed that circulating levels of IL-10 are more reduced in PD patients in comparison to healthy controls [99]. Moreover, a significantly elevated risk of progressing CP has been noted in patients with low IL-10 production [100]. IL-10, known as an anti-inflammatory regulator, blocks the production of pro-inflammatory cytokines. Additionally, IL-10 decreases the expression of major histocompatibility complex class II and co-stimulatory molecules [86]. IL-10 stimulates B cells, which strengthens B cell proliferation and differentiation. It also intensifies the secretion of anti-inflammatory mediators such as IL-1 receptor and TNF receptor antagonists [99]. Furthermore, IL-10 can regulate different subsets of CD4+ T cells, including Th17 cells and Th1 cells [101]. IL-10 plays a suppressive role in the pathogenesis of PD. Furthermore, IL-10 knockout mice have significantly higher alveolar bone loss than IL-10 competent mice after vaccination with Porphyromonas gingivalis, probably due to elevated production of pro-inflammatory cytokines [102].

Kinane et al. found no association between IL-10 gene polymorphisms and PD [103]. Yamazaki et al. [104] observed no correlation between PD patients and IL-10 polymorphisms in the gene-promoter regions. Moreover, the Japanese population had haplotype frequencies quite different from those noticed in studies of Chinese and Caucasian patients. Again, precaution in assessment is needed here, because the failure to detect an association cannot be claimed as evidence of no relationship between IL-10 and PD.

Toker et al. [105] observed that patients with the IL-10 gene −597AA genotype have a high susceptibility to CP. Similarly, Sumer et al. [106] noticed that the IL-10 gene polymorphism at position −597 seems to be connected with severe generalised CP. Chatzopoulos et al. showed that IL-10 gene polymorphisms are connected with an elevated risk of further disease progression and the necessity for further non-surgical treatment [95]. Taiete et al. indicated that the SNP rs6667202 in the IL-10 gene is associated with aggressive periodontitis in the Brazilian population. The minor C allele was less frequently found in aggressive periodontitis patients than in healthy subjects, suggesting that this SNP may have a protective effect in a Brazilian cohort [107]. In a meta-analysis, Wang et al. indicated that the −592C/A IL-10 gene polymorphism is related to PD susceptibility in Caucasians and Asians [50]. Another meta-analysis has shown that the −1082A/G polymorphism might be protective against CP in both Caucasians and Latinos, but the −819C/T and −592C/A polymorphisms might increase the risk of CP in Latinos [49].

### 2.5. Interleukin 13

Interleukin 13 (IL-13) is a pleiotropic cytokine secreted by type 2 helper T (Th2) lymphocytes [108]. IL-13 stimulates many pathological processes [109]. Additionally, this cytokine can induce inflammation, mucus production, tissue remodelling and fibrosis. IL-13 has become a therapeutic aim for many diseases, including asthma, idiopathic pulmonary fibrosis, ulcerative colitis, and other IL-13 over-expressing diseases [110]. IL-13 can have an impact on bone resorption by influencing the bone protection factor system. Researchers have discovered that PD in the gingival tissue is connected with a significant elevation in IgE and IL-13. Chen et al. suggested that the IL-13 gene polymorphism rs2243248 and haplotypes C-G-T and C-T-T may be related to CP susceptibility in the Han Chinese population [111]. In a meta-analysis including five case-control studies with 710 patients with PD and 671 healthy controls, Zhang et al. found that the IL-13 −1112C/T gene polymorphism is associated with susceptibility to chronic periodontal disease but not with aggressive periodontitis [51].

### 2.6. Interleukin 16

Interleukin 16 (IL-16) has an anti- and pro-inflammatory influence via CD4+ cell chemotactic results and the secretion of other pro-inflammatory factors [112]. Moreover, it has been observed that IL-16 has a regulatory impact on immune responses and the pathogenesis of chronic inflammatory diseases [113]. IL-16 has an influence on the immune system by altering T lymphocytes and regulating monocyte/macrophage function [113,114,115]. Monocytes play an especially crucial role in the destructive processes of PD [116], and the monocytic secretion of IL-1 and TNF-α is associated with PD [117]. However, Folwaczny et al. [118] observed no relevant association between *IL-16* gene polymorphisms and PD in Caucasians. Similarly, Vahabi et al. observed no association between the *IL-16* gene −295T/C polymorphism and PD in the Iranian population [8].

### 2.7. Interleukin 17

Interleukin 17 (IL-17) is one of the best-studied cytokines in immunology, at least in part because of its involvement in the pathology of inflammation [119,120,121,122,123]. IL-17 family cytokines are vital in host defence against microbial organisms and they play crucial roles in the pathogenesis of chronic inflammatory diseases [124]. In vivo models indicate that IL-17A is involved in pathogenesis of PD [125]. IL-17A stimulates TNF-α and IL-1β and increases the expression of other pro-inflammatory mediators [126,127]. Moreover, IL-17A influences bone remodelling through the induction of RANKL [128]. Therefore, IL-17A may be involved in the process of the destruction of the periodontal tissues [129,130]. IL-17 expression has been found in the gingival tissues of PD patients [124]. IL-17 may increase osteoclastogenesis by inducing RANKL in osteoblastic cells [131]. Similarly, the amount of IL-17 in gingival crevicular fluid (GCF) [132] and serum IL-17 levels [133] has been reported to be significantly higher in PD patients. The impact of IL-17 on chemokine secretion by osteoblasts has significant implications in the aetiology of inflammatory bone disease, including PD [134]. IL-17 is known to have the ability to initiate a severe inflammatory response involving other cytokines, chemokines and MMPs [135,136,137].

Cifcibasi et al. [123] observed that the levels of two pro-inflammatory cytokines, IL-17 and IL-23, and the neutrophil enzyme myeloperoxidase are significantly higher in AgP patients than in healthy controls. Essential decreases in these levels, both locally and systemically, were noted after therapy, possibly showing specific roles for these mediators in the active inflammation of periodontal tissues. In addition, Batool et al. noticed that salivary levels of IL-6 and IL-17 were significantly higher in patients with CP compared to healthy controls; these levels increased with the progression of CP [138]. Furthermore, Shindo et al. [139] found that IL-35 might block progression of periodontitis by reducing the IL-17A-induced overexpression of IL-6 and IL-8. Saraiva et al. examined the associations between IL-17A (rs2275913) and IL-17F (rs763780) gene polymorphisms and chronic and aggressive periodontitis [52]. The authors suggest the protective role of IL-17A (rs2275913), -197A allele against PD development, in the Brazilian population. The results of study by Chaudhari et al. suggest that IL-17A (rs2275913), -197A allele, is associated with increased risk of chronic and localized aggressive periodontitis in the Indian population [53]. The meta-analysis by da Silva et al. indicated a non-significant association between IL-17A gene rs2275913 and IL-17F gene rs763780 polymorphisms and chronic and aggressive periodontitis [140].

### 2.8. Interleukin 18

Interleukin 18 (IL-18) belongs to the IL-1 family of cytokines. It is a pro-inflammatory cytokine that plays a crucial role in PD [141]. IL-18 has anti-infection, anti-tumour and anti-hypersensitivity effects, and is related to the development of inflammatory lesions in tissues and the presence of autoimmune diseases [142]. One of the main activities of IL-18 is to stimulate the production of interferon-γ in the occurrence of IL-12 and to promote Th1 and Th2 responses [143]. IL-18 induces the secretion of other pro-inflammatory cytokines, such as TNF-α, IL-1β and IL-8, which enhance the expansion, migration, and activation of neutrophils during infection [144]. Furthermore, IL-18 has the property of inhibiting bone resorption. Therefore, it is believed that IL-18 may contribute to the regulation of bone metabolism in PD [145]. In addition, IL-18 might have an impact on periodontal health and disease through different roles, including signalling pathways and the inhibition of bone resorption [133,146].

IL-18 may have a significant role in the progress of chronic periodontitis. Mahajani et al. found that IL-18 levels in GCF were higher in patients with periodontal diseases than in healthy subjects [147]. Wang et al. found that the expression of IL-18 in the serum of patients with chronic periodontitis was increased. Additionally, the expression of IL-18 in saliva was elevated with a rising degree of periodontal destruction. Moreover, the researchers observed that IL-18 can initiate NF-κB signalling to promote the release of matrix metalloproteinases: MMP1, MMP2, MMP3 and MMP9, which may have a role in inducing the progression of chronic periodontitis [142]. Martelli et al. examined the associations between IL-18 gene −607C/A and −137G/C polymorphisms and the risk of aggressive and chronic periodontitis. The results suggest an association between these polymorphisms and chronic and aggressive periodontitis [54]. In the meta-analysis, Li et al. found a significant association between the IL-18 gene −607A/C and −137G/C polymorphisms and increased risk of periodontitis [55]. IL-18 gene −607A/C and −137G/C polymorphisms were also linked with susceptibility to aggressive periodontitis in the Uyghur population [148].

### 2.9. Interleukin 23

Interleukin 23 (IL-23) is the cytokine secreted by macrophages and dendritic cells after encountering microorganisms [149]. IL-23 is involved in expansion of the Th17 lineage, which is linked with many immune-related diseases [150]. Ohyama et al. hypothesised that IL-23 plays a crucial role in periodontal pathology. Their results reveal that IL-23 was expressed at significantly higher levels in periodontal lesions than in control sites [132]. Cifcibasi et al. observed increased levels of IL-23 in serum and GCF of patients with aggressive periodontitis compared to healthy controls [123]. Sadeghi et al. noted that the crevicular concentrations of IL-23 were lower in patients with aggressive and chronic periodontitis than in healthy subjects [137]. The results of the study by Saraiva et al. indicate a lack of statistically significant association between the *IL-23*R (rs11209026) gene polymorphism and aggressive and chronic periodontitis [52]. The current findings suggest that IL-23 plays a role in chronic periodontitis. However, further investigations with a larger population and more sensitive methods are required to explain the specific contribution of IL-23 to the pathogenesis of aggressive periodontal diseases [151].

### 2.10. Interleukin 35

Interleukin 35 (IL-35) is an anti-inflammatory cytokine created from a heterodimer of Epstein–Barr virus-induced gene 3 (EBI3) and IL-12p35 subunits [152]. IL-35 is produced by regulatory B cells, CD8+ regulatory T cells (Treg) and CD4+ regulatory T cells [150] and suppresses Th17 cell activation and IL-17 production by Th17 cells [20]. IL-35 may play a significant role in chronic inflammatory disorders such as periodontitis. However, there is limited information regarding the exact mechanisms [153].

IL-35 has been detected in GCF and in gingival tissue in CP [139]. Studies have revealed that IL-35 takes part in regulating the progression of PD by inhibiting inflammatory cytokine production [154]. Kalburgi et al. [155] were the first researchers that compared IL-35 levels in the gingival tissues of healthy controls and patients with chronic and aggressive periodontitis. The results show that IL-35 mRNA was expressed in the gingival tissues of all the three groups, but patients with chronic periodontal disease exhibited the highest IL-35 expression [156].

Jin et al. [157] noted that the expression of IL-35 protein was elevated in tissues from CP patients compared with those of healthy controls. IL-35 may block the progression of periodontal disease and thus indirectly slow systemic disease as well [158]. IL-35, as a Treg-specific suppressor of inflammatory cytokines, may keep the balance between bacterial infection and the immune system in patients with chronic periodontal disease [159]. Moreover, one of the studies assessed the expression of IL-23 and IL-35 in GCF and serum of patients with CP and compared their levels with healthy subjects. It was found that the GCF and serum levels of both cytokines were elevated in CP patients [154]. A recent research study by Kaustubh et al. has demonstrated the suppressive role of IL-35 in gingival inflammation and in maintaining periodontal health [156]. Furthermore, Raj et al. indicated that IL-35 concentrations were increased in GCF in patients with CP [153]. A recent study has revealed that IL-35 produced by regulatory T cells might block the development of periodontitis by reducing IL-17A-induced IL-6 and IL-8 expression [153]. It is still unclear whether IL-35 intensifies or antagonises the influence of other cytokines or immune cells in CP [157].

## 3. The Associations between Cytokine Gene Polymorphisms and the Immune Response to Bacterial Infection in Periodontal Tissues

Pathogens can directly damage periodontal tissue and indirectly induce the host immune response. The immune response in periodontal tissue is characterized by the infiltration of lymphocytes producing proinflammatory cytokines, enhancing the inflammatory status. The presence of bacterial infection with the increased production of proinflammatory cytokines, chemokines and matrix metalloproteinases induces progression of periodontal disease and alveolar bone loss [158]. The inter-individual differences in the inflammatory response to bacterial infections in periodontal tissue are regulated by environmental and genetic factors. The genetic factors can influence the inflammatory response to bacterial colonisation in periodontal tissue, the activation of immune cells involved in the inflammatory status, the synthesis of pro-inflammatory mediators, as well as the activation of innate and adaptive immunity [32]. The response of the immune system to bacterial infection causes the production of pro-inflammatory cytokines, which induce the differentiation, maturation, and proliferation of specific cells involved in the inflammatory status [32]. The studies have suggested that cytokines and their genetic polymorphisms may influence the immune response to bacterial infection in periodontal tissues.

Gonçalves et al. examined the associations between IL-1α +4845C/T and IL-1β +3954C/T gene polymorphisms with the subgingival microbiota and periodontal status of HIV-infected Brazilian individuals [159]. The authors observed no significant associations between IL-1 gene polymorphisms and the subgingival microbiota and periodontal status. Nibali et al. investigated the relation between IL-6 gene polymorphisms and bacterial colonisation in patients with periodontitis [160]. The IL-6 gene −174 polymorphism was associated with increased risk of Aggregatibacter actinomycetemcomitans and Porphyromonas gingivalis colonisation. The authors of another study have shown that P. gingivalis, in patients with chronic periodontitis, induces the production of IL-6, which was significantly increased in carriers of the IL-6 gene −174G allele [161]. Borilova Linhartova et al. examined the associations between IL-1, IL-6, IL-10, IL-17A, and IL-18 gene polymorphisms and the bacterial colonisation in patients with aggressive periodontitis [162]. The authors found that patients with the IL-10 −1087GG genotype have increased IL-10 plasma levels, as well as IL-10 expression in peripheral blood monocytes stimulated in vitro by periodontal bacteria [162]. In another study, these authors indicated that the release of IL-17 by mononuclear cells stimulated by periodontal bacteria was increased in carriers of the IL-17A −197A allele [163]. Krátká et al. analysed the associations between IL-1α and IL-1β gene polymorphisms and bacterial colonisation in periodontal tissue [164]. No significant connections between studied polymorphisms and bacteria composition were found. Inchingolo et al. suggested a relationship between IL-10, IL-1 and TNF-α gene polymorphisms and bacterial infections in patients with chronic periodontitis [165].

## 4. Cytokines and Age-Related Alveolar Bone Loss

Cytokines may also play a role in the pathogenesis of age-related alveolar bone loss [166]. Streckfus et al. have shown increased alveolar bone loss in postmenopausal women on estrogen therapy [167]. In addition, these women had increased salivary IL-6 concentrations compared to premenopausal women. Alayan et al. indicated that Th1 (IL-12p40, IFN-γ, TNF-α) and Th2 (IL-10, IL-4) cytokines play an important role in maintaining age-dependent alveolar bone homeostasis in mice [168]. In the study on an animal model, age-related alveolar bone loss was associated with increase in CD4^+^ T cells and increased expression of IL-17A and RANKL [169].

## 5. Therapeutic Strategies in Periodontitis

Motivations of patients to remove supragingival dental biofilm and change their behaviour for oral hygiene improvement play an important role in the treatment of periodontitis. This treatment includes supragingival professional mechanical plaque removal (PMPR), risk factor control (e.g., plaque-retentive factors, smoking, diabetes) and subgingival biofilm and calculus control by subgingival instrumentation. In this step of periodontitis therapy, adjunctive antiseptics can be used such as chlorhexidine (mouth rinses) and locally administered sustained-release chlorhexidine or specific antibiotics as an addition to mechanical debridement-subgingival instrumentation in periodontal patients. The most commonly used antibiotics are doxycycline, minocycline, amoxicillin and metronidazole. Therapy with doxycycline reduces mediators of inflammation including IL-1, IL-6, IL-17, and matrix metalloproteinases 8 and 9 [170].

In young adults with generalized stage III periodontitis, the adjunctive use of specific systemic antibiotics may be considered. The third step of periodontitis treatment may include repeated subgingival instrumentation with or without adjunctive therapies and periodontal surgery (access flap, resection, or regenerative surgery). New therapeutic strategies for PD include inflammation inhibitors, the caspase 1 inhibitor NLRP3, inflammation activation inhibitors and P2X7 antagonists [171,172,173,174,175,176,177]. These therapies not only improve clinical parameters but also influence cytokine levels.

## 6. Conclusions

PD is a chronic disease, affecting about 10% of the population. Numerous factors, such as smoking and drinking habits as well as weight gain, have been shown to be closely related to PD development [178]. The pathogenesis of periodontal disease is not yet fully understood. Patients can react in a different manner to common environmental factors. This response is affected by the individual’s genetic profile. Gingivitis will develop in many individuals with PD, but this progression is regulated by the subject’s host response. Moreover, the host response is determined by acquired immunity, but is mainly impacted by the person’s genetic profile. Individuals react to various antigens in ways predicted by their genes. There is an ongoing search to define the complex interactions of all factors that predispose individuals to PD. Additionally, more comprehensive research needs to be undertaken to reveal the most appropriate strategies for the diagnosis and prognosis of oral/systemic disorders.

## Figures and Tables

**Table 1:** Cytokine gene polymorphisms associated with periodontal disease.

Polymorphism	Study
IL-1α −889C/T; rs1800587 (5_prime_UTR_variant) T-risk allele	Feng et al. [40] (meta-analysis) 1356 patients and 1249 controls African, European and American populations
IL-1β +3953C/T; rs1143634 (Exon) T-risk allele	Parkhill et al. [41] (candidate gene study) 70 patients with and 72 controls Caucasian population
IL-1β +3953C/T; rs1143634 (Exon) T-risk allele	Da Silva et al. [39] (meta-analysis) 4924 patients and 4452 controls Caucasian and Asian populations
IL-6 −174G/C; rs1800795 (promotor) G-risk allele IL-6 −572C/G; rs1800796 (promotor) G-risk allele	Shao et al. [42] (meta-analysis) 1093 patients and 574 controls Mixed population
IL-6 −572C/G; rs1800796 (promotor) G -risk allele	Holla L et al. [43] (candidate gene study) 148 patients and 107 controls Caucasian population
IL-6 −572C/G; rs1800796 (promotor) G- risk allele	Nibali et al. [44] (candidate gene study) 224 patients with aggressive periodontitis and 231 controls Caucasian population
IL-8 −251A/T; rs4073 (promotor) T- risk allele IL-8 +396G/T; rs2227307 (intron) T- risk allele	Zhang et al. [45] (candidate gene study) 400 patients and 750 controls Asian population
IL-8 −845T/C; rs2227532 (promotor) C- risk allele	Sajadi et al. [46] (candidate gene study) 65 patients and 55 controls Iranian population
IL-8 +396 G/T; rs2227307 (intron) T-risk allele	Scarel-Caminaga et al. [47] (candidate gene study) 223 patients and 270 controls Brazilian population
IL-8 −251A/T; rs4073 (promotor) T- risk allele	Andia et al. [48] (candidate gene study) 181 patients and 108 controls Brazilian population
IL-10 −1082A/G; rs1800896 (promotor), G -risk allele in Caucasian and Latinos IL-10 −819C/T; rs1800871 (promotor), T- risk allele in Latinos	Zhang et al. [49] (meta-analysis) 3487 patients and 4356 controls Mixed population
IL-10 −592C/A; rs1800872 (promotor), A-risk allele	Wang et al. [50] (meta-analysis) 2714 patients and 2373 controls Caucasian and Asian populations
IL-13 −1112C/T; rs1800925 (promotor) T- risk allele	Zhang et al. [51] (meta-analysis) 710 patients and 671 controls Mixed population
IL-17 −197G/A; (rs2275913) (Upstream Variant) G-risk allele	Saraiva et al. [52] (candidate gene study) 90 patients and 90 controls Brazilian population
IL-17 −197G/A; (rs2275913) (Upstream Variant) A-risk allele	Chaudhari et al. [53] (candidate gene study) 35 patients with aggressive periodontitis, 35 patients with chronic periodontitis and 35 controls Indian population
IL-18 −607C/A; (rs1946518) (promotor) C-risk allele IL-18 −137G/C (rs187238) (promotor) C-risk allele	Martelli et al. [54] (candidate gene study) 213 patients and 100 controls Italian population
IL-18 −607C/A; (rs1946518) (promotor) C-risk allele IL-18 −137G/C; (rs187238) (promotor) C-risk allele	Li et al. [55] (meta-analysis) 576 patients and 458 controls Mixed population
